# The ‘goodness-of-fit’ of fit models: creating a multidimensional survey for person-organisation and person-group fit in health care

**DOI:** 10.1186/s12874-020-01033-8

**Published:** 2020-06-05

**Authors:** J. Herkes, L. A. Ellis, K. Churruca, J. Braithwaite

**Affiliations:** Centre for Healthcare Resilience and Implementation Science, Australian Institute of Health Innovation, Macquarie University, Level 6, 75 Talavera Rd, Macquarie Park, Australia

**Keywords:** Person-organisation fit, Person-group fit, Organisational culture, Workplace culture

## Abstract

**Background:**

Person-environment fit, which examines the individual’s perceptions of if, and in what way, he or she is compatible with aspects of the work context, offers a promising conceptual model for understanding employees and their interactions in health care environments. There are numerous potential ways an individual feels they “fit” with their environment. The construct was first noted almost thirty years ago, yet still remains elusive. Feelings of fit with one’s environment are typically measured by surveys, but current surveys encompass only a subset of the different components of fit, which may limit the conclusions drawn. Further, these surveys have rarely been conducted in a focused way in health care settings.

**Method:**

This article describes the development of a multidimensional survey tool to measure fit in relation to the person’s work group (termed person-group (P-G) fit) and their organisation (person-organisation (P-O) fit). The participants were mental health care employees, volunteers, and university interns (*n* = 213 for P-O fit; *n* = 194 for P-G fit). Confirmatory Factor Analyses (CFAs) were conducted using LISREL.

**Results:**

Valid and reliable sub-scales were found.

**Conclusion:**

This advanced multidimensional survey tool can be used to measure P-O and P-G fit, and illuminates new information about the theoretical structure of the fit construct.

## Background

The concept of individuals’ interactions with their work environment has long captured the attention of researchers. While they can be motivating and satisfying to work in, health care settings can suffer from unhealthy localised cultures, and poor employee outcomes [1–6]. Particularly, health environments can perpetuate hierarchies, tribal behaviours, communication siloes [7], bullying and incivility [1, 2], which indicate poor organisational and workplace cultures. In health care, staff’s perceptions of their compatibility with their organisational and workplace cultures have been found to have important associations with their feelings of wellbeing, burnout, and intention to leave [8], as well as being associated with important downstream effects on patients [9] through decreased employee productivity [10], and increased risk of medical errors [11, 12]. It has been suggested that understanding organisational and workplace cultural characteristics may be important in explaining these phenomena.

Intervening in this relationship between staff and their organisation has proved challenging; there is limited understanding of how to design and implement effective cultural interventions, and as many as 70% of localised culture change interventions both in and outside of health care are thought to fail [13]. To develop more appropriate interventions, we first need to understand and appropriately measure the constructs involved. One approach to understanding the interaction between staff members and their work environment is through person-environment (P-E) fit. This is an emerging theoretical lens on how staff perceive and experience their work environment - one that is multifaceted, yet plagued by questions of definition and measurement [14, 15].

P-E fit is comprised of several distinct levels of environmental interaction, which have been typically studied independently [15–18]. However, it is beneficial to investigate multiple levels of environmental interaction simultaneously, as staff never actually experience these aspects of the environment in isolation [15, 19]. For example, staff may experience varying levels of fit with their job, their work group and their organisation. This research project, developed as part of a wider study on organisational and workplace culture, focuses on person-organisation (P-O) and person-group (P-G) fit dimensions, as these are the most commonly targeted environmental levels in culture change interventions [14, 15]. In this manuscript, the inclusion of both P-O and P-G fit in the same scale is unique, allowing greater nuance to be measured than if these elements were measured individually.

In addition to individuals interacting with different aspects of the work environment, they can experience fit differently. These components of fit, or potential ways of fitting in, are synthesized in Table 1 [14, 17]. These components are often studied individually rather than collectively, which again greatly limits the conclusions derived, because different types of fit can have variable or interacting effects on employee outcomes [8]. All of the listed components will be included in the current study.

**Table 1:** Components of fit with organisation^a^

Component of fit	Definition
Supplementary fit or similarity fit	Compatibility in which the individual and organisation are congruent [14, 20]. This component emphasizes the consistency of the person and the values, goals, and “personality” that permeate the organisational culture.
Complementary fit	Fit in which the individual or organisation fills a gap in, adds something unique to, or “makes whole” the other [20–22].
Needs-supplies fit or supplies-values fit	A feeling of fit in which the needs, inclinations or requirements of the person are fulfilled by the organisation, e.g., desire for further training or support [14, 23].
Demands-abilities fit	Fit in which the individual has the required capability and capacity to meet the demands of the organisation [14].

*Note*. ^a^The same components are hypothesised to exist for interactions between the person and their work group

**Fig. 1:**
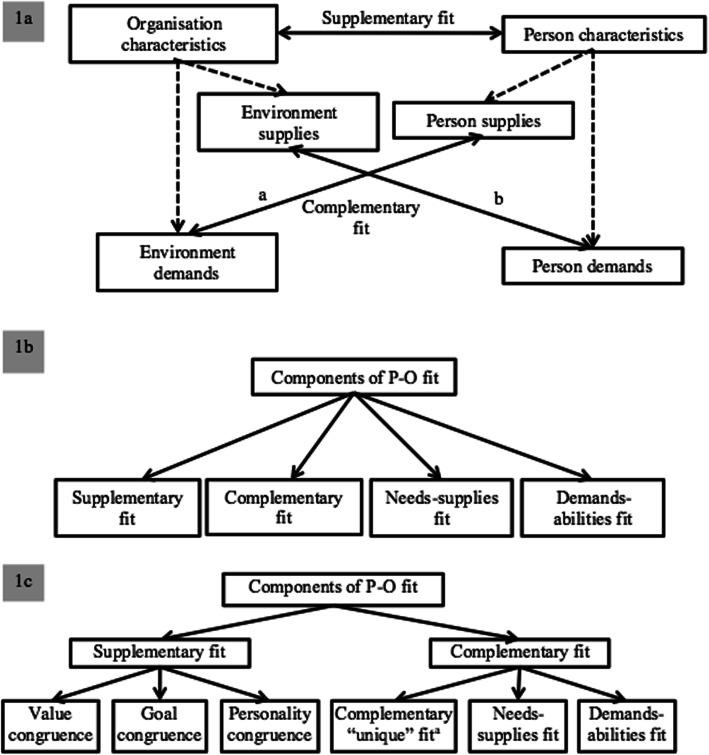
Different theoretical representations of the relationship between the components of P-O and P-G fit. 1a: Demands-abilities fit (arrow label “a”) is characterised by the person supplying what the environment demands, such as resources (time, effort and commitment) [14]. In needs-supplies fit (arrow label “b”), the environment supplies what the person demands, including resources (financial, physical and psychological) and opportunities (task-related and interpersonal) [14]. **.**1b: This school of thought measures complementary fit as a separate construct.**.** 1c: Synthesis of Fig. 1a and b. ^a^Complementary “unique” fit measures are derived from Fig. 1b. *Source*: Author’s conceptualisations, adapted from Kristof [14] (Fig. 1a), Piasentin and Chapman [20], Piasentin and Chapman [21] and Guan, Deng [24] (Fig. 1b)

There are conflicting perspectives on how these components interact with one another within P-O fit. Some researchers define needs-supplies and demands-abilities fit as sub-components of complementary fit (Fig. 1a) [14, 25], whilst others describe complementary fit as a distinct component (Fig. 1b) [20, 21, 24]. These differing schools of thought have resulted in the development of many measurement tools which are difficult to reconcile in a single study [18, 20].

The P-G fit field is even more embryonic in nature. There has been a dearth of studies that have explicitly measured P-G complementary, needs-supplies and demands-abilities fit [26, 27]. A review of research in other areas of P-E fit (e.g., P-O fit) [18, 23, 28] suggests that needs-supplies and demands-abilities fit permeate *all* levels of the environment, and so theoretically should be present in P-G fit. Furthermore, inspection of published P-G fit study tools indicates an implicit measurement of needs-supplies and demands-abilities [26, 27, 29, 30]. This suggests that the lack of survey development may be due not to the absence of these components, but rather the emergent nature of the field. All-in-all, the literature to date suggests there are sound studies of individual components of fit [14–18, 20, 21, 23–28]. What we are missing is a holistic understanding of the fit construct, and a tool to measure it. It is to the task of filling in this gap in knowledge that we now turn.

## Methods

### Aim

To resolve the ambiguity of the components encompassed in P-O and P-G fit, and to attempt to reconcile the different schools of thought, a conceptual model was developed (Fig. 1c). This model attempted to account for the complexity of the person’s experience of their environment [18]. If validated, the model has the potential to further knowledge on organisational and workplace cultures in health care. Based on this model, this article aimed to *develop and validate a holistic, multi-dimensional tool to measure P-O and P-G fit*. In line with this working model, two hypotheses (**H**) were developed. H1 focuses on P-O fit, while H2 focuses on P-G fit.

HI: *It was hypothesised that needs-supplies fit and demands-abilities fit would be sub-factors of complementary fit in the P-O fit factor structure.*

H2: *It was hypothesised that (in addition to supplementary and complementary fit), needs-supplies and demands-abilities fit would each be significant, distinct components within P-G fit.*

### Participants

Ninety-seven centres within a large, distributed health care group across Australia were invited to participate, and 31 centres across six states accepted the invitation [8, 31]. The sample size necessary for an adequately powered confirmatory factor analysis (CFA) is widely debated [32]. As the number and type of variables present in P-O and P-G fit literature is ambiguous, a numerical minimum was deemed most appropriate. Based on a commonly accepted rule-of-thumb [33], a minimum sample of 100 participants was targeted.

### Measures of P-O and P-G fit

A multi-dimensional survey tool was developed using distinct items to measure each hypothesised component of P-O and P-G fit. Many P-O fit survey questions were modified slightly for the current study [34]. P-G measures were more difficult to identify than P-O items and often required additional tailoring. Each item was rated on a seven-point Likert scale, from ‘strongly disagree’ [1] to ‘strongly agree’ [7]. The final survey questions for each component of P-O and P-G fit are provided in the **Supplementary File,** Table 1.

### Preliminary data analysis

#### Missing data

In the survey data, 15.0 and 25.6% of item results were missing for the P-O fit CFA and P-G fit CFA, respectively. Data cleansing techniques were applied to reduce bias and increase the representativeness of the sample [35, 36]. The Expectation Maximization (EM) algorithm was used to provide Maximum Likelihood (ML) estimates, offering a sophisticated and accurate data substitution technique to estimate the value of the missing data [37–39]. This EM algorithm was undertaken in IBM SPSS Version 24 [40] to compute missing values at the sub-scale level.

#### Reliability

For this study, SPSS was used to calculate Cronbach’s Alpha (*α*) to measure internal consistency and reliability [41]. Alpha values greater than 0.70 were considered as satisfactory, and 0.80 as excellent [42].

#### Factor structure

Data were imported into PRELIS and subsequently analyzed using LISREL 9.30 [43]. Multiple CFAs were conducted to test the hypotheses, including those with first- and second-order factors [44]. A number of common statistics (Table 3) were used to assess the validity of the instruments.

## Results

Data from the survey including the mean and standard deviation for each is supplied in Table 2.

**Table 2:** Descriptive statistics for fit variables

Variable (***n*** = 213 for P-O; ***n*** = 194 for P-G)	Mean	Standard deviation
P-O Value congruence	5.80	0.94
P-O Goal congruence	5.73	0.87
P-O Personality congruence	5.64	0.89
P-O Complementary fit	4.71	1.16
P-O Needs-supplies fit	5.43	1.16
P-O Demands-abilities fit	5.87	0.87
P-G Value congruence	5.55	0.91
P-G Goal congruence	5.43	1.05
P-G Personality congruence	5.52	0.90
P-G Complementary fit	5.07	1.08
P-G Needs-supplies fit	5.68	0.97
P-G Demands-abilities fit	5.87	0.77

### P-O fit CFA

**Fig. 2:**
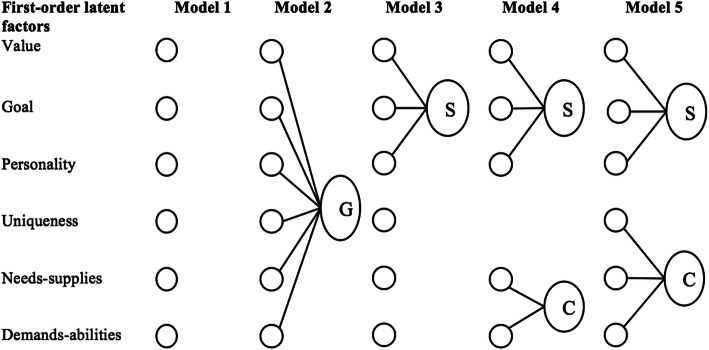
Higher-order factor structures for Models 1–5 to be tested in the P-O and P-G fit CFAs. G = General latent factor; S=Supplementary fit; C=Complementary fit; Small circles represent first-order factors; larger circles represent second-order latent factors; Model 3 and Model 4 are based on literature interpretation. Model 2 and Model 5 are the authors’ conceptualisations

The P-O CFAs (*n* = 213) were conducted in stages to identify the most suitable factor model (Fig. 2) [44]. The difference in the goodness-of-fit statistics was negligible between the first- and second-order models, suggesting parsimony [45]. Fit statistics were then used to determine which second-order model provided the best approximation of the data [45]. Model 4 was excluded based on the χ^2^/*df* ratio and its relatively high Root Mean Square Error of Approximation (RMSEA). Model 5 had a lower Akaike Information Criterion (AIC), indicating better fit than Models 2 and 3, and thus was deemed the most acceptable model (Table 3) [45]. Thus, the results supported H1 as the model with the best goodness-of-fit matched to the hypothesised working model of P-O and P-G fit.

**Table 3:** Comparison of the goodness-of-fit of P-O and P-G fit models

Model	***df***	χ^**2**^/***df***^a^	RMSEA	RFI	TLI	SRMR	AIC^b^
***Accepted Values***	N.A.	2–4	***≤***0.05	0.9–0.95	0.9–0.95	***≤***0.05	N.A.
**P-O CFA results**							
*Model 1*	120	2.695	0.0892	0.856	0.905	0.052	6647.587
Model 2	129	2.709	0.090	0.856	0.904	0.063	6655.734
Model 3	126	2.971	0.096	0.842	0.889	0.121	6686.598
Model 4	84	4.011	0.119	0.832	0.869	0.070	5932.777^g^
Model 5	128	2.613	0.087	0.861	0.087	0.056	6642.653
Modified Model 5	124	2.045	0.071	0.890	0.940	0.051	6569.868
**P-G CFA results**							
Model A	48	2.909	0.099	0.900	0.932	0.059	3798.971
Model B	50	3.037	0.102	0.895	0.927	0.064	3807.188
Model C	50	3.037	0.102	0.895	0.927	0.064	3807.188
Modified Model B	48	2.635	0.092	0.909	0.942	0.058	3785.819
Modified Model C	49	2.854	0.098	0.902	0.934	0.063	3797.192

*Note*. *RMSEA* Root Mean Square Error of Approximation, *RFI* Relative Fit Index, *TLI* Tucker-Lewis Index; χ^2^ = chi-square; SRMR = Standardized Root Mean Square Residual; AIC = Akaike Information Criterion, which compares second-order non-nested models, lower scores indicate better fit

^a^Model 1 χ^2^ = 323.34; Model 2 χ^2^ = 349.48; Model 3 χ^2^ = 374.35; Model 4 χ^2^ = 336.89; Model 5 χ^2^ = 334.40; Modified Model 5 χ^2^ = 251.46; ^†^Model A χ^2^ = 139.61; Model B χ^2^ = 151.83; Model C χ^2^ = 151.83; Modified Model C χ^2^ = 139.83

^b^The AIC of Model 4 cannot be compared to the other models as there is one less first-order latent variable. AIC of Models A-C were added for completeness, but are not compared

The goodness-of-fit for Model 5 was further improved through alteration of modification indices that, where theoretically justifiable, were entered sequentially into the a-priori CFA. Item pairs on the same target factor only were modified, and the largest modification indices were freed first. Alterations included freeing the error covariance between POV2 and POV3; POG2 and POG4; PON2 and PON3; and POD2 and POD3. Ultimately, this CFA yielded a χ^2^ of 251.46 (df = 124), a Tucker-Lewis Index (TLI) of 0.940, Relative Fit Index (RFI) of 0.890, Root Mean Square Error of Approximation (RMSEA) of 0.071, and Standardized Root Mean Square Residual (SRMR) of 0.0508. The high covariance between second-order latent variables (.83) suggested that both sub-scales were indeed part of the same P-O fit scale. Ultimately, the goodness-of-fit statistics provided moderate support for the psychometric strength of the P-O fit factor structure. Thus, H1 was accepted.

### P-G fit CFA

**Fig. 3:**
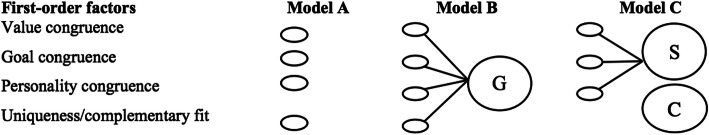
Second-order models to be tested in the P-G CFA. S=Supplementary fit; C=Complementary fit; G = P-G general factor. The small circles are first-order latent factors, and the larger circles are second-order latent factors

As with P-O fit, the first-order P-G fit model was first established (*n* = 194). However, unlike P-O fit, multiple first-order models were tested as there was less of a theoretical basis for which first-order model was most appropriate. The most appropriate first-order model (Model A, Fig. 3) did not include needs-supplies or demands-abilities items. Two second-order factor models (Model B and C) were then tested for parsimony with Model A.

Models B and C had comparable goodness-of-fit statistics, including TLI and RFI, making it difficult to determine the model of best fit. However, on examination of the residual variances (which, for second order factors, represent the proportion of the true score variance that cannot be explained by higher order factors) [46], it appeared that modified Model C accounted for slightly more of the true scores for the items than modified Model B. Furthermore, the additional latent factor in modified Model C compared to modified Model B accounted for the slightly inflated AIC value. Thus, modified Model C was selected as the most appropriate model, providing the most theoretically nuanced version of the data. The error variances were freed to improve the model, where theoretically justifiable (e.g., PGG1 and PGG2 was freed on Model C to create modified Model C; see Table 3).

Acceptable values for the statistics were based on peer-reviewed literature. RFI and TLI values guided by Byrne [47], χ2/df ratio from Marsh and Hocevar [44], RMSEA and SRMR from Steiger [48]; Hu and Bentler [49], Hooper, Coughlan [50].

### Residual variances analysis

Sum scores were created through averaging the survey responses across each item. No reverse coded questions were included in the final survey. The average percentage of variance of the items explained by these factors is 63%. In all of the factors, with the exception of item POD2 (error variance = 0.56), the second-order factor score explained more than half of the true score variance, which was deemed exceptional [46]. In the P-G fit CFA, the residual error variances of modified Model C indicated that the second-order factor of complementary fit accounted for 62% of the true scores in P-G complementary fit items, and the supplementary fit second-order factor accounted for 73% of the variance in value, goal and personality congruence items. Moreover, none of the residual error variances were over 0.40, indicating that the model was exceptional at accounting for item variance (**Supplementary File,** Table 2). This suggested that, although the fit statistics themselves were modest, the model rigorously accounted for the variance of first-order factors.

### Reliability

**Fig. 4:**
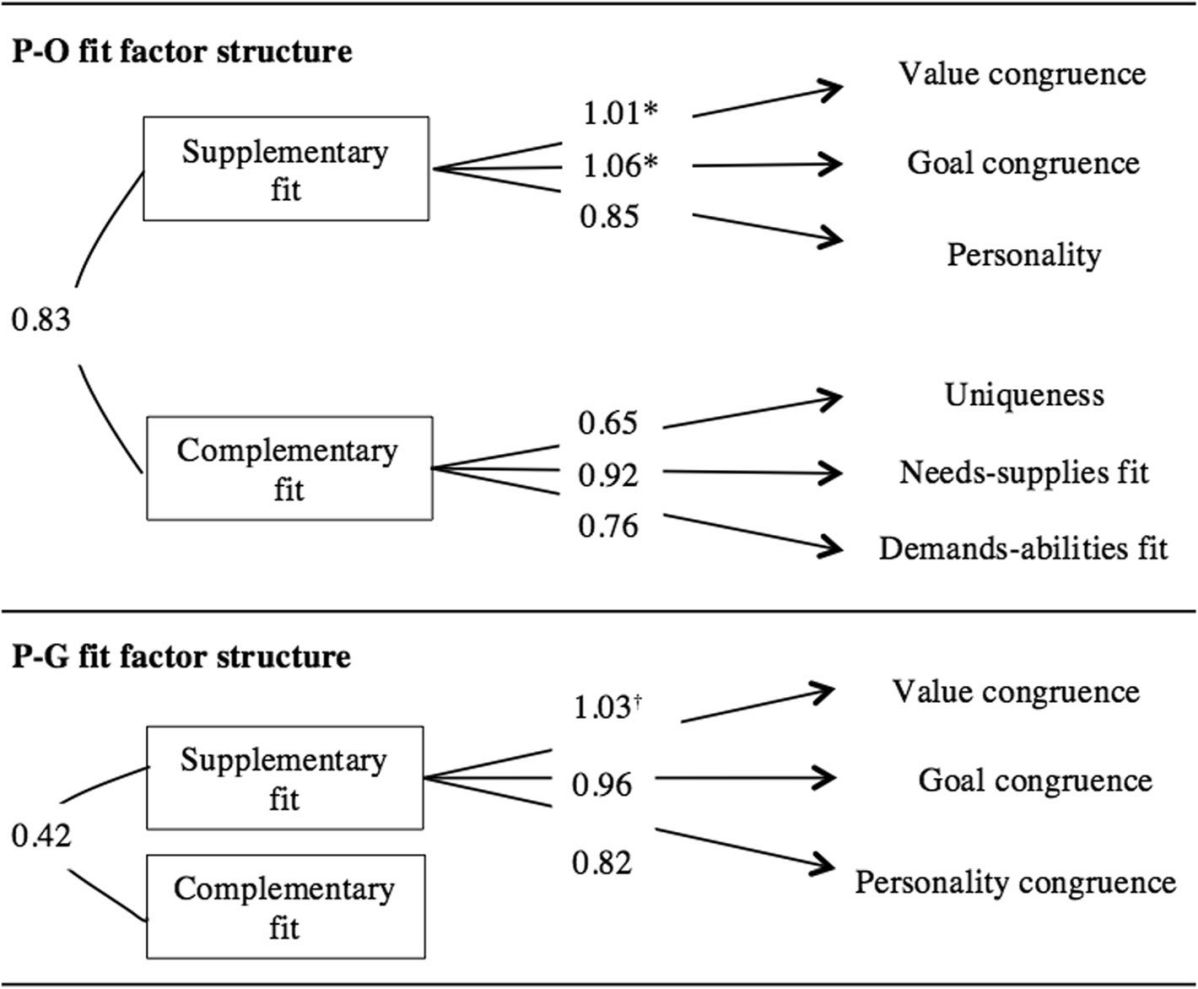
Second-order P-O and P-G fit factor structures. Each of the first-order factors consists of three items. ^†^As explained by Jöreskog, standardised coefficients can be above a magnitude of 1 [51].

Internal consistency estimates of the first- and second-order latent factors were examined for the P-O and P-G fit CFAs (Table 4; Fig. 4). Estimates ranged from satisfactory to excellent (.77 to .92) for the P-O fit CFA, and good to excellent (range = .80 to .93) for the P-G fit CFA.

**Table 4:** Reliability statistics for latent factors

P-O factors		Cronbach’s alpha	Mean inter-item correlation	Number of items
2nd order factors	Supplementary fit	.921	0.614	9
	Complementary fit	.845	0.619	9
	**Mean reliability score**	.883		
1st order factors	Value congruence	.857	.669	3
	Goal congruence	.807	.586	3
	Personality congruence	.809	.588	3
	Uniqueness	.770	.528	3
	Needs-supplies	.890	.737	3
	Demands-abilities	.814	.594	3
	**Mean reliability score**	.825		
**P-G factors**				
2nd order factors	Supplementary fit	.926	.799	9
	Complementary fit	.796	.379	3
	**Mean reliability score**	.861		
1st order factors	Value congruence	.849	.658	3
	Goal congruence	.812	1.042	3
	Personality congruence	.869	.697	3
	**Mean reliability score**	.843

### Synthesis of reliability and CFA results

A final analysis was completed on both P-O and P-G fit items together. Based on published surveys in the literature that have measured multiple sub-scales of P-E fit in the one study, there has been no final CFA conducted including all sub-scales [15]. Rather, only the correlations amongst the measures have been reported. Corresponding with previous research, the correlations amongst the ten factors in this study are presented (Table 5**),** with the highest correlations between P-O value and goal congruence (r = 0.82), and the same components at the P-G value and goal congruence (r = 0.81). Conceptually, these high correlations were explained by previous research that has often grouped and validated the association between aspects of supplementary fit [14]. More importantly, the low correlations between the items in different CFAs (P-O factors versus P-G factors) suggested satisfactory discrimination between the factors of the different sub-scales.

**Table 5:** Correlations amongst the 10 factors

	POV	POG	POP	POC	PON	POD	PGV	PGG	PGP	PGC
**POV**	1.0									
**POG**	0.817	1.0								
**POP**	0.648	0.619	1.0							
**POC**	0.350	0.427	0.431	1.0						
**PON**	0.633	0.659	0.588	0.436	1.0					
**POD**	0.431	0.493	0.473	0.352	0.550	1.0				
**PGV**	0.504	0.518	0.491	0.384	0.367	0.353	1.0			
**PGG**	0.380	0.485	0.356	0.221	0.340	0.320	0.806	1.0		
**PGP**	0.373	0.367	0.582	0.283	0.279	0.297	0.763	0.686	1.0	
**PGC**	0.176	0.244	0.227	0.434	0.173	0.259	0.374	0.338	0.377	1.0

*Note*. POV=P-O value congruence; POG = P-O goal congruence; POP=P-O personality congruence; POC=P-O complementary/uniqueness items; PON=P-O needs-supplies fit; POD = P-O demands-abilities fit; PGV=P-G value congruence; PGG = P-G goal congruence; PGP=P-G personality congruence; PGC=P-G complementary/uniqueness fit

Ultimately, the factor structure of each instrument was identified. Consistent with H1, the factor structure of P-O fit was found to include all identified a-priori factors in the hypothesised latent structure. The goodness-of-fit indices for each model suggested reasonable fit, and the items had consistently high factor loadings. H2 was partially supported, as the best CFA model of P-G fit included only four of the six hypothesised latent components. However, when this was tested psychometrically, there was found to be a good fit of the model. The factor correlations also showed satisfactory discrimination between the scales.

For each item, internal consistency reliability estimates were good, with the possible exception of Uniqueness in the P-O fit scale and Complementary fit in the P-G fit scale, which both scored acceptable reliability. Thus, the results demonstrated that the sub-scales were reliable measures of fit.

## Discussion

This study aimed to develop a holistic, multi-dimensional tool to measure P-O and P-G fit not previously provided. The results provide unique insights into the underlying components of fit and how they affect each other in a health care context. The adequate goodness-of-fit and reliability attained for the second-order P-O and P-G fit models adds to the past literature, suggesting that perhaps the two schools of thought in fit literature may be integrated rather than viewed as two different paradigms.

The findings from the P-O CFA adds to previous fit literature, as both Model 3 and 4, which correspond to different conceptualisations within past literature (Fig. 1b and a respectively), had acceptable fit statistics [14, 20, 21, 24, 25]. Neither model yielded fit statistics that surpassed those of Model 5, which the research team developed based on a synthesis of Model 3 and Model 4 (see Fig. 1c). This suggests that there is an alternative to researchers subscribing to one of the two complementary fit schools of thought, as this third model could provide an opportunity for researchers to explore P-O fit more holistically. Hence, these findings contribute to a deeper understanding of P-O fit and specifically in a health care context.

The findings from the P-G CFA results are commensurate with previous literature [14], which validates that these factors manifest in health care. The needs-supplies and demands-abilities questions did not adequately fit the factor structure to be included in the final factor model. The omission of these components from the factor structure in this study suggests that further work is needed to develop and test items that adequately capture these hypothesised components of P-G fit [26, 27], or may open the possibility that these constructs are different at this level of environmental interaction.

The CFAs produced reliable and valid sub-scales for assessing P-O and P-G fit, which are particularly suitable for use in health care. These measures may act as a foundation for future research into the experience of fit, so that the survey tools are more aligned with the theoretical models in this field.

### Implications for health care

There is increasing research highlighting an association between the organisational culture of a health service and patient outcomes [9], which suggests a positive effect of P-O (and to a lesser extent P-G) on staff outcomes [8]. As part of this growing area of interest, the survey validated here can be used to better understand organisational and workplace cultures in health care and beyond to make decisions to improve the wellbeing of their employees (e.g., improving alignment between their employees and their organisation). In health care, the untapped potential of leveraging the influence of organisational and workplace cultures could benefit not only the employees, but also the patients. This can be achieved by recognising and harnessing the cultural risk and protective factors for staff and patient outcomes [52, 53].

### Strengths and limitations

One strength of the study is the inclusion of all theorised elements of P-O and P-G fit, not just those that had been previously widely measured. Because of this, the survey offers a foundation for future research in the P-E fit paradigm. Limitations included the relatively small sample size for CFA analysis which, when combined with having just-identified latent factors, may have decreased the goodness-of-fit for both models [54]. Although the goodness-of-fit statistics of the models were acceptable, they did not fulfil the strict criterion of the most conservative cut-off values for excellent factor structure [44, 46]. Future research with a more conservative CFA sample size, and including other types of health professionals, should take this into consideration and develop further items for each latent factor to minimise the effect of this limited sample size.

## Conclusion

Addressing the limitations of past literature, multi-dimensional survey sub-scales were developed for this study, which included more aspects of P-O and P-G fit than have been included in previous surveys. In a study in mental health care, the survey tool was validated through multiple CFAs, and the reliability of its sub-scales was verified. This is an important stepping-stone for future research into P-O and P-G fit, especially in health care. Although further research is recommended—on P-G fit in general and the components of needs-supplies and demands-abilities fit, in particular—the results of this article contributed a new, unique understanding of the nuanced theoretical framework of P-O and P-G fit.

## Supplementary information

**Additional file 1: Supplementary File 1.** Includes Table 1. Original fit survey items and their corresponding hypothesised latent. Factors; and Table 2. P-G and P-O fit CFA included items statistical information and factor Loadings.

## Data Availability

The datasets analysed during the current study are available in the Supplementary file and throughout the manuscript. The datasets used during the current study available from the corresponding author on reasonable request.

## Abbreviations

AIC: Akaike Information Criterion
CFA: Confirmatory factor analysis
P-E: Person-environment
P-G: Person-group
P-O: Person-organisation
RFI: Relative Fit Index
RMSEA: Root Mean Square Error of Approximation
SRMR: Standardized Root Mean Square Residual
TLI: Tucker-Lewis Index
χ^2^: Chi-square
