# Supplementary material for: The ‘goodness-of-fit’ of fit models: creating a multidimensional survey for person-organisation and person-group fit in health care
Source: BMC Med Res Methodol. 2020 Jun 5;20:144. doi: 10.1186/s12874-020-01033-8 (PMC7275356; doi:10.1186/s12874-020-01033-8)
Supplement: Supplementary file 1 — Additional file 1: Supplementary File 1. Includes Table 1. Original fit survey items and their corresponding hypothesised latent. Factors; and Table 2. P-G and P-O fit CFA included items statistical information and factor Loadings. [file 12874_2020_1033_MOESM1_ESM.pdf]

Supplementary File:

Table 1 provides the original fit survey, including original items, and maps them to their corresponding latent factors. Note that the final survey item codes are listed in the article. **Table 2** provides information about the included statistical items and factor loadings.

Table 1. Original fit survey items and their corresponding hypothesised latent factors

Component identified		Type of fit being measured (code)	
		**P-O**	**P-G**
Supplementary fit	Value	• “The things that I value in life are very similar to the things that my organisation values”[1] (POV1)<br>• “My personal values match my organisation’s values and culture”[1] (POV2)<br>• “My organisation’s values and culture provide a good fit with the things that I value in life”[1] (POV3)	• “The things that my work group members value in life are very similar to my values”[2]† (PGV1)<br>• “My values match my work group’s values”[2]† (PGV2)<br>• “My work group's values provide a good fit with the things that I value in a group”[2]† (PGV3)
	Goal	• “I identify strongly with the goals of my organisation”[3] (POG1)<br>• “My personal goals and the goals of my organisation are very similar”[3] (POG2)<br>• "I **don't** care about the goals of this organisation as much as the organisation would ideally like me to"[3]† *(POG3)<br>• "My goals match or fit those of the organisation"[4]† (POG4)	• “The people in my work group and I have the same expectations of the effort needed to reach goals”[5]† (PGG1)<br>• “My work group have the same expectations of reward for goal achievement as I do”[5]† (PGG2)<br>• “My goals are similar to those of the people in my work group”[6]† (PGG3)

Personality	• “My personality matches or ‘fits’ this organisation”[7]† (POP1)<br>• “The ‘personality’ of the organisation reflects my own personality”[7]† (POP2)<br>• “My personality is well suited for the personality or ‘image’ of this organisation”[8] (POP3)<br>• "My personality prevents me from fitting into this organisation because it is different from other employees' personalities" [8]†* (POP4)	• “I feel that my personality matches my work group’s image”[9]† (PGP1)<br>• “My personality matches my work groups’ personality”[9]† (PGP2)<br>• “My personality provides a good fit with my work group’s personality”[9]† (PGP3)
General/<br>Unique complementary fit	• “I feel that I am a unique piece of the puzzle that makes this organisation work”[8] (POC1)<br>• “My organisation seem to value that I am different from the ‘typical’ employee”[8]† (POC2)<br>• “My knowledge, skills, and abilities offer something that other employees in this organisation do not have”[8] (POC3)	• “When key decisions are made, people in my work group consult me because I have a different perspective than they do”[8]† (PGC1)<br>• “Even though my personality differs from the people in my work group it seems to complement their personalities”[8] (PGC2)<br>• “I feel that I am an important part of my work group because I have such different skills and

	• "I feel like I stand out in this organisation"[8] (POC4)	abilities than the people in my work group"[8] (PGC3)<br>• "The people in my work group rely on me because I have competencies that they do not have"[10] (PGC4)
Needs-supplies fit	• "There is a good fit between what headspace offers me and what I am looking for in an organisation"[1]† (PON1)<br>• "The attributes that I look for in an organisation are fulfilled very well by headspace"[1]† (PON2)<br>• "Headspace gives me just about everything I want from an employing organisation"[1]† (PON3)	• "I feel that my work group understand and listen to me"[11]† (PGN1)<br>• "My work group provides me with a variety of interesting things to do"[12]† (PGN2)<br>• "I personally see opportunities to learn new things from my work group"[13]† (PGN3)<br>• "I get the help and support I need from the people in my work group"[12]† (PGN4)
Demands-abilities fit	• "The match is very good between the demands of my organisation and my personal skills"[1]† (POD1)<br>• "My abilities and training are a good fit with the requirements of my organisation"[1]† (POD2)<br>• "My personal abilities and education provide a good match with the demands that	• "My abilities and training are a good fit with the requirements of my work group"[2]† (PGD1)<br>• "My knowledge, skills and abilities provide a good fit with the needs the work group have of me"[2]† (PGD2)<br>• "The match is very good between the demands of my work group and my skills"[2]† (PGD3)

		my organisation places on me”[1]† (POD2)

Note. †Original wording of the survey item altered for the current study. The reference after each item indicates the source upon which each particular survey item was based.

Participants will be asked to rate their agreement with each item on a seven-point Likert scale, from strongly disagree to strongly agree.

Consistency was obtained between items e.g., replacing the word “company” with “organisation” and “team” with “work group”.

To apply these items to other organisations, supplement “headspace” with the organisation name of the study setting.

*Reverse scored.

Table 2. P-G and P-O fit CFA included items statistical information and factor loadings

P-O CFA			Mean	S.D.	Residual variance	Factor Loading
Supplementary fit	Values	POV1	5.68	1.09	0.26	0.86
		POV2	5.88	0.99	0.38	0.79
		POV3	5.83	1.08	0.31	0.83
	Goals	POG1	5.86	0.97	0.47	0.73
		POG2	5.44	1.22	0.42	0.76
		POG4	5.69	1.03	0.43	0.76
	Personality	POP1	5.66	1.08	0.31	0.83
		POP2	5.16	1.23	0.37	0.79
		POP3	5.74	1.09	0.46	0.73
Complementary fit	Uniqueness	POC1	4.97	1.50	0.43	0.75
		POC2	4.83	1.50	0.30	0.83
		POC4	4.18	1.42	0.54	0.68
	Needs-supplies	PON1	5.63	1.21	0.14	0.93
		PON2	5.55	1.19	0.33	0.82
		PON3	5.10	1.45	0.26	0.86
	Demands-abilities	POD1	5.76	1.05	0.22	0.89
		POD2	5.97	0.99	0.56	0.66
		POD3	5.87	1.02	0.49	0.71
**P-G CFA**						
Supplementary fit	Values	PGV1	5.40	1.11	0.38	0.79
		PGV2	5.62	0.97	0.22	0.88

		PGV3	5.63	1.04	0.14	0.93
	Goals	PGG1	5.42	1.32	0.40	0.77
		PGG2	5.28	1.26	0.39	0.78
		PGG3	5.59	1.08	0.33	0.82
	Personality	PGP1	5.55	0.96	0.21	0.89
		PGP2	5.40	1.12	0.26	0.86
		PGP3	5.62	0.94	0.12	0.94
Complementary fit	Uniqueness	PGC1	4.89	1.46	0.44	0.75
		PGC3	5.09	1.41	0.30	0.84
		PGC4	4.99	1.55	0.40	0.77

Note. Statistics reported to two decimal places; Factor loading is standardised.

S.D – standard deviation.
